# Theoretical and Practical Principles on Nanoethics: A Narrative Review Article

**Published:** 2019-10

**Authors:** Saeed BIROUDIAN, Mahmoud ABBASI, Mehrzad KIANI

**Affiliations:** 1. Department of Medical Ethics, School of Traditional Medicine, Shahid Beheshti University of Medical Sciences, Tehran, Iran; 2. Ethics and Law Research Center, Shahid Beheshti University of Medical Sciences, Tehran, Iran

**Keywords:** Nanomedicine, Nanoethics, Comparative study

## Abstract

**Background:**

Interests in nanotechnology and its application in medical research, diagnosis, and treatment of diseases continuously grow. The study identified the theoretical and practical principles of ethics in developed countries' nanomedical research to be used as the first step of development of a national nanoethics standard or guideline in Iran and developing countries.

**Methods:**

The present study was done between 2012–2016 in Ethics and Law Research Center, Shahid Beheshti University of Medical Sciences, Tehran, Iran, which comprised a literature review and a comparative study to describe and compare the nanoethics situation and considerations of nanoethics in Australia, Canada, and USA.

**Results:**

The main ethical considerations in the three countries contain two major categories, including firstly, the nature of nanoparticles such as its diversity, rapid development of new and not well-defined nanoproducts and particles and unpredictable side effects of such nanoparticles; and secondly, the application of developed nanoparticles in areas such as justice, privacy protection, patient-physician relations, etc.

**Conclusion:**

It is controversial to develop an independent nanoethics standard or codes; however, national priorities and concerns, as well as specific nanoethics considerations, should be investigated before deciding to create such standards in each country. Overall, careful considerations have to take into account the justice, privacy protection, the inherent risks of nanomaterials and their possible side effects on patients and other study subjects, as well as considering characteristics of new developed nanoproducts and particles.

## Introduction

Interest in nanotechnology and its application in medical research, diagnosis, and treatment of diseases are on a constant growth trend. Every day, a range of novel nano drugs, nano equipment and nanobiochips is introduced to the global market (1, 2). Despite the benefits of such progress, nanomedical technology, like any other technology, is accompanied by a number of consequences (2). As the intersection of engineering, physics, biology, medicine, and chemistry, nanotechnology facilitates the application and empowerment of these sciences in the production of innovative products. Following the substantial progress of nanotechnology research over the past decade, increasing attention has been devoted novel drugs, drug delivery systems and treatment methods for diseases such as cancer to promote human health through nanotechnology (3, 4).

The term “*nanotechnology*” is composed of the unit prefix “*nano*” (derived from a Greek word-meaning dwarf) which means one billionth and the word technology. Nanoscience deals with phenomena, properties, and interactions of various substances at the atomic, molecular, and macromolecular levels (sizes of 1–100 nm) where, especially at sizes smaller than 5 nm, materials manifest completely different characteristics compared to larger scales. Nanotechnology is hence the design, manipulation, and production of nanoscale (< 100 nm) equipment and systems through controlling the shape, size, properties, reactions, and performance of structures (5).

The European Science Foundation has defined nanomedicine as a branch of science involving the application of molecular knowledge and instruments to relieve pain and enhance human health by the prevention, diagnosis, and treatment of diseases and traumatic injuries (6, 7).

Nanomedicine is the use of nanoscale approaches, theories, equipment, and structures to detect, prevent, or treat various illnesses by the identification and restoration of damaged tissues at the molecular level. In addition to its advantages in pharmaceutics, nanomedicine also seeks to provide advanced diagnostic pathology and treatment services at the molecular or even micro-molecular level. Attention to such consequences of nanomedicine is critical since this field of science deals directly with human health (8).

### Ethics in Nanomedicine

Nanoethics is a set of ethical principles used in all nanotechnology-based research and diagnostic and therapeutic measures. It is a branch of bioethics, which is in turn categorized under professional ethics (9, 10).

To describe better the concept of nanoethics in the context of medical ethics, it is necessary to know more about ethical concerns in nanomedicine.

Although nanomedicine has provided potential solutions to many long-standing unsolved medical problems, the ethical issues related to its emergence have been commonly neglected. Since ethical considerations have not developed at a pace similar to the science itself, investments are required to fill the gap between science and ethics (9, 11, 12). Raj Bawa and Summer Johnson (pioneers of discussions on social and ethical aspects of medicine) have highlighted investments on nanomedicine in pharmaceutical industry. Pharmaceutical companies' desire to maximize their profit and their consequent tendency toward investments in nanotechnology might prevent them from paying sufficient attention to social and ethical considerations (13).

While subjects such as human dignity, autonomy, and secrecy are indispensable to any biological study, they are even more prominent in nanomedicine research due to the nature of nanotechnology. As discussed earlier, nanomedicine is a branch of modern technology, which incorporates rapid developments and productions to facilitate the prevention, diagnosis, and treatment of diseases at cellular and biomolecular levels. Nevertheless, owing to the unclarity and unpredictability of long-term effects of nanomedicine, this field of science cannot be compared to any other branches of technology when ethical issues are concerned. In other words, despite the startling achievements of nanomedicine during the short period since its emergence, the obscurity of its long-term effects increases the significance of ethics in this field. Despite its undeniable benefits to modern medicine, nanomedicine (like any other field of research) has raised particular legal, social, and ethical issues whose neglect would lead to major crises in the future (11,12).

### Nanotechnology in Iran

First sparkles of nanotechnology in Iran were lit in 2001, and two years after the beginning of the studies, “Iran Nanotechnology Initiative Council” was established in 2003 with direct support of parliament and presidency (14). Iran Nanotechnology Initiative Council created various workgroups for the development of nanotechnology such as Nanotechnology Development Workgroup, Human Resources Development Workgroup, Technology Infrastructures Workgroup, Public promotion and Culture-making Workgroup, etc. to perform a 10 yr strategic plan in Iran. After a decade of huge investment in nanotechnology, now, according 2016 statistics, Iran has more than 28000 researchers and 14000 Master and PhD students and graduates in this field, ranked 7th in the world in the number of Nano-articles in ISI and 9th in the citations, and first in Nanotechnology new method patents in USPTO (15).

Considering the dramatic development of nanotechnology in Iran, “Iran Nanotechnology Safety Network” was established in 2012. The aim of the network is to define national standards, regulations, and instructions on safety, environmental and ethical issues in nanotechnology.

The Ethics Committee of this network decided to provide a national standard for nanoethics to be considered in nanotechnology research and products (16).

During last two decades, some national guidelines of ethics in medical research have been developed (17); however, since nanomedicine is a novel technology in Iran's medical sector, no particular ethical considerations and codes have been developed to deal with the ethical issues arising from nanomedicine research. The identification and localization of the ethical policies adopted by pioneers of nanotechnology would hence minimize the adverse consequences of nanotechnology, especially nanomedicine, research and practice in Iran.

Therefore, the aim of this study was to investigate the status of nanoethics such as ethical considerations in nanomedicine in Australia, Canada, and the US as three pioneers in nanoethics as the first step to be able to define and develop a national standard for nanoethics in Iran. In fact, this comparative study is the first phase of this guideline/standard development in cooperation of “Iran Nanotechnology Safety Network”. Therefore, the present study sought to identify and summarize the theoretical and practical principles and considerations of ethics in nanomedical research in three pioneers of nanomedicine (Australia, Canada, and the US) in the light of a comparative study to be used later in development of a national standard/guideline.

## Methods

This comparative study has described and compared situation and considerations of nanoethics in Australia, the US, and Canada as pioneers of nanoethics. Comparative research is a research methodology in which differences and similarities between phenomena are identified through comparisons between countries, cultures or other sectors. It is widely applied in macro sociology to answer questions at the intermediate and macro levels using internal and external analysis. (18)

Therefore, the research comprised two distinct levels. At the first level, a literature review was conducted to describe and illuminate the existing status of nanoethics considerations. To this purpose, scientific databases, including PubMed, Scopus, and Web of Science, were searched using nanoethics, nanotechnology, nanomedicine, medical ethics, and ethics in nanotechnology as key terms. The next stage involved a comparative study of the status of nanoethics in, Australia, Canada, and USA. These three countries were selected due to their attention to medical ethics, as well as their keen interest and massive investments in nanomedicine. The available books, article, and online databases were again searched (with a number of key terms including applications of nanotechnology, nanomedicine, nanoproducts, medical ethics, and ethical codes in nanomedicine) to extract relevant information in each country.

An internal analysis was performed on the extracted information to determine the ethical standards governing nanomedicine studies, along with their strengths and weaknesses, in the target countries and to evaluate such standards based on the cultural context of each society. The ethical aspects of nanomedicine studies were first described in the target countries. The obtained data were further analyzed and summarized with the comparative approach. Finally, the findings of both levels and the ethical considerations in each country were meticulously analyzed.

## Results

The present study compared three countries in terms of ethical considerations in nanomedicine. The objective was to facilitate the establishment of ethical codes in nanomedicine research in Iran through the identification of the applied approached and developed ethical codes in the mentioned countries. The following sections will thus describe the findings of the comparative study.

### Findings in Australia, Canada, and the US

Medical research and practice are deals directly with human health. As nanotechnology is a novel technology whose consequences cannot be easily predicted due to its nanoscale nature, the existing ethical codes do not seem to suffice. The subject of nanoethics is of particular significance in Iran, a country that is still a novice in this field despite its progress in the number of scientific articles and production of science. In order to facilitate the development of relevant ethical considerations and codes in the country, Table 1 summarizes the major ethical considerations in Australia, Canada, and the US.

**Table 1:** Nanoethical considerations in Australia, Canada, and the US

***Country***	***Nanoethical considerations***	
Australia	Justice (19)	Nano-gap between developed and developing countries or between the poor and the rich in developed countries
	Privacy protection (20)	The risk of accidental disclosure or unethical use of confidential information
	Patient-physician relations (19, 21)	- Diagnosis overtakes treatment. - Development, ease of use, and concurrence with information technology may raise an issue about the safety of tests, their reliability under uncontrolled conditions, and effects of diagnosis without access to consultation and support.
	Increased capabilities of humans (22–25)	Nanotechnologies may soon provide people with opportunities to change their appurtenance, performance, or even character. Ethical issues related to such advances should be taken into account.
Canada	Nano-gap (26, 27)	Uncertainty about the fair distribution of benefits and identification of risks associated with the use of nanotechnology.
	Ethics in biomedical research (26, 28)	The ethical concerns about the use of nanotechnology in the health sector are similar to those regarding the use of other emerging technologies in biomedical research.
	Ethics in diagnostic and therapeutic applications (27–29)	- Predicting the potential health and environmental risks of nanotechnology requires a link between various fields of science. - The diversity of the possible applications of nanotechnology and the subsequent concerns necessitates the explanation of the resultant ethical issues. - There are concerns about the affordability of nanodrugs in the public health system.
The US	Unclarity of the side effects of nanoparticles (30,31)	Are the effects of nanoparticles in medical and environmental applications limited to the target fields?
	Diversity of nanomaterials (30,31)	Despite the diversity of nanomaterials, little research has evaluated their application. Studies on one particular nanomaterial cannot be generalized to other materials.

Ethical considerations in these three countries are summarized in different codes. Each code is more described in literature related to each country. For example, Justice and nano-gap (in Australia and Canada) are described more as followed below. The other items of table are discussed in discussion section of this paper.

Justice as an ethical principle in bioresearch implies that all people should receive equal health facilities regardless of their personal characteristics such as ethnicity, gender, or age (32).

Nanotechnology is predicted to develop to more than two-trillion-dollar industry embracing advanced pharmaceutical products, improved medical imaging techniques, more precise surgical instruments, and unpredictable medical breakthroughs (33). Since such progress might be limited to countries with considerably high gross domestic products (at trillion-dollar levels), a wide gap, called a nano-gap, may be created between developed and developing countries (34, 35). Developed countries would be more capable of producing and expanding nanotechnologies and benefiting from their production. Moreover, researchers may solely focus on introducing innovative nanotechnological solutions to the problems faced by developed countries. Developing countries would hence be forced to resolve their health and environmental issues through more sustainable, less technology-dependent strategies. In the absence of adequate resources to run their own research projects, these countries would have to rely on research findings of developed countries (34, 36).

There are also raising concerns about intellectual property rights in nanotechnology, i.e. patents on nanotechnology might encompass large numbers of applications and thus restrict access to information and technology, especially in developing countries. The nano-gap might also broaden the gap between the rich and the poor in developed countries and make people access to different levels of novel technologies, particularly medical advances, based on their wealth (34,36).

## Discussion

The increasing application of nanotechnology and nanomedicine warrants attention to ethical issues in this field. Hence, the present study sought to redefine the theoretical and practical principles of ethics in nanomedicine in Iran and three pioneers of nanotechnology (Australia, Canada, and the US).

Ethics in nanomedicine refers to the ethical issues involved in the use of nanotechnology in medical research and development of diagnostic and therapeutic measures. It is a branch of bioethics, which can be categorized, in turn, under professional ethics. In the three studied countries, the main ethical considerations in nanomedicine can be classified into two major categories. The first group of concerns related to the nature, diversity, and unpredictable side effects of nanoparticles. The second group stemmed from the application of these materials can be summarized as:

- Uncertainty about the fair distribution of the benefits of nanotechnology: The benefits of nanotechnology might be exclusively available to developed countries (and increase the dependence of other nations on these countries) or even worse, to the upper classes of their societies.
- The risk of disclosure of confidential information: Nanotechnology might be able to access people's personal information without their consent. This would definitely jeopardize privacy protection (one of the most important ethical issues in medicine).
- Transformation of patient-physician relationships: The achieved progress may lead to the diagnosis of currently incurable diseases. Being diagnosed with an untreatable disease would lead to profound psychological consequences for the patients and their families. Furthermore, knowledge about the existence of incurable diseases (e.g. AIDS) causes a great level of public anxiety. On the other hand, patients may become able to diagnose and treat their own diseases. The possibility of self-diagnosis and self-treatment will result in disastrous social consequences. For instance, in case of diseases with overlapping symptoms, self-treatment may prevent timely and accurate diagnosis of the real disease and cause serious problems in the patient.
- Increased capabilities of humans: Nanotechnology may provide individuals with the chance to modify their appearance, performance, and even personality. Such possibilities would indubitably raise major ethical issues. This is particularly important when cloning and its consequent concerns, e.g. violation of human dignity, identify challenges, and interference in the laws of nature, are involved.
- Ethics in Biomedical Research: Since general research in nanotechnology involves human trials, issues related to privacy protection, autonomy, and human dignity are of critical significance in this field. In fact, the unclarity and unpredictability of the possible consequences of nanomaterials may affect the processes of information provision, obtaining informed consent, and privacy protection in nanomedicine research.
- Ethics in Diagnostic and Therapeutic Applications: Considering the increasing attention to the use of nanotechnology in the diagnosis and treatment of various diseases, the possible adverse effects of such measures on human health and the environment need to be thoroughly evaluated. Moreover, the ethical issues rising from the possible future applications of nanotechnology should be clearly described.

Due to the absence of a global standard or code of ethics in nanomedicine, developing a comprehensive ethical code would require an interdisciplinary study to identify the different aspects of ethical and social consequences of nanomedicine. This study provides an overview and summarized considerations of nanoethics in three studied countries. Our findings could be used as an overview as the first step to develop a national standard/guideline in Iran and also other countries more involved in nanomedicine.

## Conclusion

Although some new ethical aspects and different considerations are mentioned in nanomedicine area, but there is a high overlap between common ethical principles of medical ethics and nanoethics; therefore, it is a controversy about development of a new independent field or codes of nanoethics according to previous studies and an independent ethics principles for nanoethics is needed. However, more studies are needed for each country to consider its own context, nanotechnology situation, national needs, concerns, and national medical ethics codes before deciding to develop any national nanoethics codes or standards.

## Ethical considerations

Ethical issues (Including plagiarism, informed consent, misconduct, data fabrication and/or falsification, double publication and/or submission, redundancy, etc.) have been completely observed by the authors.
